# Oncologic standards in colon cancer resection: from margins to lymph node yield and mesentery

**DOI:** 10.3389/fsurg.2026.1831262

**Published:** 2026-06-22

**Authors:** Mariarosaria Portinaio, Carlo Alberto Schena, Michele Ammendola, Geoffrey Yuet Mun Wong, Patricia Tejedor, Fausto Rosa, Elisa Reitano, Corrado Pedrazzani, Jim Khan, Nicola de’Angelis

**Affiliations:** 1 Department of General Surgery, Fondazione Poliambulanza Hospital, Brescia, Italy; 2 Operative Unit of Digestive Surgery, University “Magna Grecia”, Catanzaro, Italy; 3 Department of Upper Gastrointestinal Surgery, Royal North Shore Hospital, Sydney, NSW, Australia; 4 Colorectal Surgery Unit, University Hospital Gregorio Marañón, Madrid, Spain; 5 Università Cattolica del Sacro Cuore, Rome, Italy; 6 Department of Emergency and Trauma Surgery, Fondazione Policlinico Universitario A. Gemelli IRCCS, Rome, Italy; 7 Department of Surgery, Strasbourg’s Nouvel Hôpital Civil (NHC), Strasbourg, France; 8 Division of General Surgery, Department of Surgical Sciences, Dentistry, Gynecology and Pediatrics, University of Verona, Verona, Italy; 9 Department of Colorectal Surgery, Portsmouth Hospital University and NHS Trust, Portsmouth, United Kingdom; 10 University of Portsmouth, Portsmouth, United Kingdom; 11 Department of Translational Medicine, University of Ferrara, Ferrara, Italy

**Keywords:** central vascular ligation, colon cancer, D3 lymphadenectomy, lymph node yield, mesocolic excision, resection margins

## Abstract

**Background:**

Oncologic quality in colon cancer surgery hinges on achieving adequate longitudinal margins, lymphadenectomy, and mesenteric excision, yet international standards and segment-specific practices remain heterogeneous.

**Methods:**

A focused PubMed/MEDLINE search from inception to October 2025 identified international guidelines, randomized and non-randomized comparative studies, large registries, and meta-analyses addressing: (1) longitudinal resection margins; (2) lymph node (LN) yield and nodal metrics; and (3) complete mesocolic excision (CME) with central vascular ligation (CVL)/D3 versus standard colectomy in curative-intent colon cancer surgery. The study aimed to delineate current standards in colon cancer resection.

**Results:**

Mapping studies and guidelines converge on 5–7 cm longitudinal margins for most tumors, with extension toward 10 cm in advanced T stage (cT3–cT4 disease) or when arterial geometry indicates a longer at-risk pericolic segment. LN assessment beyond the historical ≥12 minimum improves staging accuracy and survival, with several large datasets suggesting a plateau around 18–22 examined nodes. CME/CVL/D3 reliably increases nodal yield and specimen quality without excess morbidity in optimized settings and appears to confer an oncologic advantage across stages II–III, with the most consistent survival signal in stage III disease.

**Conclusion:**

An oncologically adequate colectomy should aim for: (1) R0 resection with 5–7 cm margins, extendable toward 10 cm when dictated by tumor stage or vascular anatomy; (2) mesenteric resection to the feeding-vessel origin targeting 18–22 LNs (minimum ≥12), with systematic reporting of tumor deposits and LN ratio; and (3) thoughtful use of CME/CVL/D3 embedded within structured training and routine audit of outcomes, particularly for stage III right colon cancers.

## Introduction

Oncologic quality in colon cancer surgery has come to the forefront in recent years with standardized perioperative pathways and new pathological targets. Regardless of surgical approach, the essential goal of colon cancer surgery is the *en bloc* removal of the primary tumor and its regional lymphatic basin with intact mesocolic planes, achieving an R0 resection and an adequate lymphadenectomy to allow accurate staging and regional disease control. These technical endpoints extend beyond local control, influencing long-term outcomes, the use and appropriateness of adjuvant chemotherapy, and healthcare resource burden (1–3). Indeed, inadequate resections risk understaging and undertreatment, while excessive dissection may increase morbidity without clear oncologic benefit if performed indiscriminately.

Over recent decades, the dissemination of complete mesocolic excision (CME), central vascular ligation (CVL), and D3 lymphadenectomy, together with the widespread adoption of minimally invasive and robotic techniques, has reshaped how an “adequate” colectomy is defined. Surgical quality, defined by margins, nodal clearance, and plane fidelity, remains paramount. However, several regional differences persist, and controversies are observed worldwide concerning the definition of oncological standards for colectomy.

This narrative review revisits the concepts underpinning an oncologically adequate colectomy and evaluates resection margins, lymphadenectomy adequacy, and CME/D3/CVL effectiveness in light of recent studies and guideline updates.

## Methods

This narrative review sought to answer the following question: “What are the current oncologic standards for colon cancer resection?”. A literature search was performed on PubMed/MEDLINE from inception to October 1, 2025, using the following keywords and/or MeSH terms: *colon cancer* AND *resection margins* OR *lymphadenectomy* OR *central vascular ligation* OR *D3* OR *complete mesocolic excision* OR *lymph node yield* OR *extended resection* OR *standard resection*. International guidelines, consensus statements, randomized and non-randomized comparative studies, prospective and retrospective cohorts, and systematic reviews/meta-analyses were analyzed. Priority was given to primary sources and high-quality registries. Adult patients with colon adenocarcinoma undergoing curative-intent resection comprised the population of interest. Rectal-specific studies were excluded unless directly informative for colon segments. Outcomes of interest were resection margins, lymph node (LN) yield, and CME/D3/CVL. Given heterogeneity, a qualitative narrative synthesis was conducted, reporting directions of evidence and areas of consensus or uncertainty.

## Results

In order to reduce locoregional recurrence and distant metastases, colonic resections for colon cancer must comply with oncologic principles, such as appropriate resection margins, adequate LN harvest, and completeness of mesocolic excision. Strict adherence to these standards is essential for improving disease-free survival (DFS) and overall survival (OS).

1. What is the optimal length of proximal and distal resection margins required to ensure oncologic adequacy in colon cancer surgery?

Guidelines from Japan, the United States, and Europe do not fully converge on optimal longitudinal resection margins. Longitudinal margins should encompass the pericolic lymphatic basin draining along the arterial arcade that supplies the tumor. The extent of resection is tied to the definition of pericolic LNs, which should always be removed due to their metastatic potential, and to the level of vascular ligation.

The Japanese Society for Cancer of the Colon and Rectum (JSCCR) links the at-risk pericolic field to the territory of the feeding artery rather than a fixed metric and classifies pericolic LN fields based on artery-tumor geometry as D1 (pericolic nodes), D2 (intermediate nodes along the feeding artery), and D3 (apical nodes at the vascular origin) (4). In clinical practice, D3 dissection is recommended for cT3–cT4 lesions and may also be considered for cT2 tumors (at least a D2 dissection is required for cT2 cancer), and longitudinal margins are anchored to the arterial territory of the feeding vessel (4). Furthermore, the Japanese Classification of Colorectal Carcinoma, categorizes pericolic LNs into four types, determined by their position relative to the tumor and the feeding artery (5, 6): a) If the feeding artery is situated directly beneath the tumor, the affected zone reaches 10 cm in both the oral and anal directions from the tumor's edge; b) Should a single feeding artery be located within 10 cm of the tumor margin, the region stretches 5 cm past the point of arterial inflow, and the opposing side extends 10 cm from the tumor boundary; c) In cases where two dominant arteries are present within 10 cm of the tumor margin, the area expands 5 cm beyond each arterial inflow site, both orally and anally; d) If no feeding arteries are found within 10 cm of the tumor margin, the affected zone reaches 5 cm past the nearest artery to the tumor margin, while the opposite side extends 10 cm from the tumor boundary.

Mapping studies show that metastatic pericolic nodes cluster within 5–7 cm from the tumor edge, and involvement beyond 10 cm is rare. In a series of 164 patients, Hida et al. stratified resections by tumor-artery distance and suggested that 3 cm margins may be adequate for T1 lesions, 5 cm for T2, and 7 cm with central lymphadenectomy for pT3-pT4, with 96.9% of node-positive cases contained within 7 cm when central lymphadenectomy is performed (7). Ueno et al. corroborated this distribution in a multicenter cohort of 2,996 patients with stages I-III colon cancer, reporting pericolic metastases mostly within 7 cm and only exceptional spread beyond 10 cm (0.1%), supporting the 10-cm rule as a practical upper bound for the regional pericolic field (8). Huang et al. analyzed 1,958 patients and showed that shorter distal margins (<3 cm) were associated with higher anastomotic recurrence (probably due to intraluminal exfoliated tumor cells or micrometastases), whereas margins ≥6 cm reduced this risk (9). Molecular data support pro-carcinogenic signaling gradients in peritumoral tissues that decline over the first centimeters from the tumor, especially in advanced disease (10).

The 2022 American Society of Colon and Rectal Surgeons (ASCRS) guidelines recommend 5–7 cm proximal and distal margins with mesenteric resection to the origin of feeding vessels, ensuring removal of intermediate and central nodes, with en bloc resection for adjacent organ invasion (T4b) (11). Similarly, the 2020 European Society for Medical Oncology (ESMO) guidelines advise margins of at least 5 cm on either side of the tumor with *en bloc* mesentery resection and en bloc resection for adjacent organ invasion (T4b) (12).

Segment-specific considerations matter: right-sided tumors often have wider pericolic basins along long right colic/middle colic arcades; transverse and flexure tumors may receive dual inflow with crossover drainage; sigmoid lesions typically map along the inferior mesenteric/left colic territory, where mesenteric length is constrained by arterial geometry.

*Practical take-home message: A target of 5*–*7 cm proximally and distally captures the vast majority of at-risk pericolic nodes; extension toward 10 cm can be reasonable in cT3*–*cT4 tumors or when arterial geometry suggests a longer at-risk segment, provided anastomotic perfusion and functional length are preserved.*

2. Is a lymph node yield of at least 12 nodes adequate to ensure accurate staging and oncologic quality in colon cancer surgery?

The historical benchmark of ≥12 nodes originated to ensure adequate staging [specifically to identify Dukes' C colon cancers (13)], especially the detection of node-positive disease, rather than as a therapeutic threshold. Contemporary evidence consistently associates higher LN counts with improved survival, likely reflecting a combination of stage migration, more complete regional clearance, and biological correlates of host-tumor interaction. Major Western guidelines, namely the American Joint Committee on Cancer (AJCC), the National Comprehensive Cancer Network (NCCN), and ESMO, still recommend a minimum of 12 nodes for accurate staging, whereas JSCCR guidelines tie extent to D1-D3 rather than a fixed numeric threshold based on clinical staging (4, 11, 14, 15). In JSCCR guidelines, D1 lymphadenectomy is considered sufficient for pTis tumors, D2 is indicated for pT1 tumors, and D3 for tumors classified as cT3–cT4. For cT2 cancer, at least a D2 dissection is required, although a D3 dissection may also be considered.

### Key comparative and cohort evidence supporting lymph node yield thresholds

Large cohorts and registry analyses show better OS with ≥12 nodes examined and suggest incremental benefits up to approximately 18–22 nodes, beyond which survival gains may plateau.

Mroczkowski et al. analyzed 7,012 patients with stage II–III colon cancer stratified by lymph node yield (<12 vs ≥12). Five-year overall survival was higher when ≥12 nodes were examined (68% vs. 63%; *p* = 0.027). The study also highlighted the independent prognostic value of the lymph node ratio (LNR) and noted that higher yields were more common in right-sided cancers. A key limitation is potential confounding from adjuvant and other therapies when isolating the effect of lymphadenectomy (16). Simoes et al. compared outcomes in patients with ≥22 nodes examined versus those meeting the conventional ≥12-node benchmark. A yield ≥22 was associated with longer DFS (adjusted HR 0.75; *p* = 0.031) and OS (adjusted HR 0.71; *p* = 0.025) across TNM stages. The authors attributed the advantage to the removal of micrometastases and tumor deposits, with the greatest benefit observed in right-sided cancers, which generally carry a poorer prognosis (17). Bundred et al., using large registry cohorts, questioned lymphadenectomy as a pure quality metric and instead framed node yield as a biological prognostic marker. Beyond roughly nine nodes, additional retrieval only marginally increased N + detection, yet each extra node corresponded to about a 1% reduction in mortality risk, consistent with improved staging accuracy and possibly a more robust antitumor immune response. The authors concluded that adequate lymphadenectomy refines prognosis and helps individualize adjuvant therapy (18). Webber et al. evaluated predictors of inadequate lymphadenectomy (<12 nodes) versus adequate dissection (12-20 nodes). Inadequate lymphadenectomy was less frequent after right colectomy and more common after left colectomy, and it was associated with worse OS. Patients with >20 nodes retrieved had better survival than those with 12-20, raising questions about whether the 12-node threshold is universally sufficient (19). Similarly, Tian et al. analyzed large SEER and external validation cohorts to estimate the optimal number of examined lymph nodes (ELNs) by N stage. They reported that increasing ELNs was associated with improved survival and reduced understaging up to an approximate plateau around 18-19 nodes overall, with stage-specific nuances; beyond this threshold, additional ELNs conferred minimal further survival benefit (20).

### Beyond node count: lymph node ratio and tumor deposits

Qualitative nodal features also matter: tumor deposits (discrete mesenteric foci without nodal architecture) portend worse outcomes independent of N category and should be reported. The LNR (positive/total nodes) further stratifies risk within the same N stage and mitigates the interpretive limitations of low total counts.

Men et al. assessed the relationship between node count and 5-year mortality and evaluated the prognostic role of LNR. While supporting ≥12 nodes for accurate staging, they found no additional staging benefit beyond 22 nodes. Nonetheless, yields of about 20-22 nodes were associated with significantly lower 5-year mortality (per-node HR approximately 0.985). The authors suggest that incorporating LNR into TNM could improve prognostic precision (21). Addressing limitations of the TNM nodal category, Kim et al. showed that tumor deposits are independent adverse prognostic factors even in the presence of nodal metastases. Although the AJCC (8th ed.) defines N1c only when no positive nodes are present, their findings indicate that tumor deposits portend worse outcomes regardless of nodal involvement (22). In alignment with Japanese practice, Marunaka et al. did not endorse a fixed numeric node target. Instead, they recommended D1, D2, or D3 dissection tailored to tumor stage and proposed a Lymphadenectomy Index, calculated as the frequency of metastasis at each nodal station multiplied by the 5-year OS of patients with metastases, to appraise adequacy without relying on stage-based node counts (23).

*Practical take-home message: Examination of ≥12 nodes should be considered as a minimum to avoid understaging; target 18-22 nodes is a reasonable institutional quality goal, achieved through mesocolic-plane surgery with appropriate vascular control and close surgeon-pathologist collaboration. Pathology reports should systematically document tumor deposits and LNR to complement TNM and refine adjuvant therapy discussions.*

3. Should complete mesocolic excision and central vascular ligation be preferred over standard colectomy to improve oncologic quality and lymphadenectomy?

The concepts of CME and D3 lymphadenectomy are inconsistently reported across guidelines (Table 1). Western guidelines emphasize *en bloc* mesocolic excision with adequate nodal evaluation but stop short of mandating CME/CVL in all cases. The Italian Association of Medical Oncology (AIOM) guidelines stress oncologic resection with ligation at the root of the mesocolon, while cautioning against routine high ligation at the superior mesenteric artery (SMA) and vein (SMV) during right colectomy because of vascular risks (24). According to the German S3 guidelines (25), oncologic surgery should ensure complete tumor resection combined with an adequate lymphadenectomy. These guidelines emphasize that the extent of bowel resection is mainly determined by central vascular division and removal of the corresponding lymphovascular drainage territory. Paracolic lymphatic spread rarely extends beyond 10 cm from the tumor, supporting adequate proximal and distal resection margins together with complete mesocolic excision of the tumor-bearing segment. Regarding lymph node harvest, these guidelines, in line with other European recommendations, continue to recommend the examination of at least 12 lymph nodes to ensure accurate pathological staging.

**Table 1:** Guidelines comparison.

	ESMO 2020 (15)	ASCRS 2022 (11)	JSCCR 2019 (4)	NCCN 2025 (14)
Longitudinal margins	≥5 cm proximally and distally	5–7 cm proximally and distally	Margin length is linked to the feeding-artery territory rather than a fixed distance; mapping data support a practical 10-cm upper limit for the regional pericolic field	No single fixed cm specified
LN yield	≥12 LNs	≥12 LNs; suggests removing suspicious nodes outside the standard field when feasible	No fixed numeric minimum; classifies D1-D3 levels, with extent defined by T stage and arterial territory	≥12 LNs; suggests removing suspicious nodes outside the standard field when feasible
CME	The term CME is not used. Recommend oncologic mesocolic-plane surgery with adequate lymphadenectomy	Recommend oncologic mesocolic-plane surgery with adequate lymphadenectomy; routine extended lymphadenectomy (CVL/D3) is not recommended	The term CME is not used. Recommendations are framed as D1/D2/D3 lymphadenectomy: D3 dissection is recommended for cT3–cT4 lesions (and may be considered for selected cT2)	Recommend proximal ligation of vascular pedicles with en bloc mesentery

ASCRS, American Society of Colon and Rectal Surgeons; CME, complete mesocolic excision; CVL, central vascular ligation; ESMO, European Society for Medical Oncology; JSCCR, Japanese Society for Cancer of the Colon and Rectum; LN, lymph node; NCCN, National Comprehensive Cancer Network.

ESMO recommends *en bloc* resection of the colon and mesentery to clearly distinguish stage II from stage III disease, without explicitly requiring CME or CVL (12). ASCRS and NCCN guidelines define extended lymphadenectomy as either D3 resection or CVL (removal of lymphatic tissue along the SMA and vein during right colectomy and along the inferior mesenteric artery [IMA] during left colectomy) and describe CME in terms of the completeness and integrity of the mesocolic envelope (11, 14). The Japanese framework operationalizes extent by D1-D3 based on T stage and arterial territory, as seen before (4).

Although often used together, CME, CVL, and D3 lymphadenectomy describe related but distinct elements of oncologic technique. CME is primarily a plane-based concept (sharp dissection along the mesocolic fascia to preserve an intact mesocolic envelope), CVL refers to central ligation of feeding vessels (to encompass intermediate/central nodes and maximize vessel-to-tumor distance), and D3 defines the nodal field extending to the vascular origin. In practice, these concepts overlap, yet they are not interchangeable. In right colectomy, CME is frequently combined with CVL and a D3-level dissection, but CME can also be performed without a formal D3 field or true central ligation, especially on the left and transverse colon, where vascular anatomy is more variable. This heterogeneity complicates study comparisons and meta-analyses.

Across comparative series and meta-analyses, CME and D3-level dissections produce longer resection lengths, greater vessel-to-tumor distance, larger mesenteric area, and higher LN yield, with signals toward reduced local recurrence and improved survival, particularly in stage II–III disease, without consistent increases in perioperative morbidity when performed in optimized settings (26–28). Indeed, the historical concern that D3 lymphadenectomy/CME may increase perioperative morbidity due to the more extensive central vascular dissection performed in proximity to the superior mesenteric vessels is not supported by the most recent evidence (29–32). In the meta-analysis by Xu et al. 2023 (33), which included only randomized controlled trials, CME was associated with a significant reduction in major postoperative complications compared with standard colectomy, without any increase in overall complication rates or anastomotic leakage. Furthermore, intraoperative blood loss was lower in the CME group, suggesting that dissection performed according to sound anatomical principles can be achieved safely. Similarly, Van Eetvelde et al. 2025 (30), in a European multicentre analysis of 551 patients undergoing robotic right colectomy, demonstrated that the stepwise implementation of CME did not result in higher 30-day postoperative morbidity, major complication rates, mortality, or length of hospital stay, despite increased technical complexity and longer operative times. Of particular interest was the complete absence of superior mesenteric vein injuries across the entire cohort and the lack of any increase in vascular events associated with central lymphovascular dissection. Safety is also suggested by the study by Jarry et al. (34) that evaluated the learning curve of minimally invasive CME for right-sided colon cancer, focusing on operative metrics and complications. The authors established a consistent proficiency plateau for minimally invasive CME at 21-32 cases, supporting its safety and efficacy even during initial phases. Taken together, these findings suggest that D3 lymphadenectomy/CME does not increase perioperative morbidity compared with conventional colectomy, while preserving the potential oncological advantages associated with a more extensive lymphadenectomy.

### Right-sided colon cancer

Hohenberger originally defined CME as sharp dissection in the mesocolic plane with true central ligation at the origins of the mesenteric vessels to clear the lymphatic basin while avoiding mesocolic tears (26). In this series of 1,329 R0 resections (1978–2002), the median LN harvest was 32 overall and 29 among node-negative patients. Five-year cancer-specific survival for the whole cohort was 85%, and the 5-year locoregional recurrence rate was 4.9%. Complication rates were within expected ranges for colon surgery (overall complications 19.7%). These outcomes improved over time alongside the standardization of this technique. Notably, the study was non-randomized and included only R0 resections. Across subsequent syntheses, short-term safety of CME appears comparable to standard colectomy, with suggestions of oncologic benefit but low-certainty evidence. Contemporary European Association of Endoscopic Surgery (EAES) guidelines, with the Society of American Gastrointestinal and Endoscopic Surgeons (SAGES) and European Society of ColoProctology (ESCP) participation, define CME as resection of the entire mesocolic root to the right of the SMV with central ligation of ileocolic vessels, identification of Henle's trunk, preservation of the gastroepiploic vein, and ligation of right branches of the middle colic vessels, with ≥5 cm colonic margins (27). Experts suggested similar perioperative mortality, major intraoperative bleeding, and major postoperative complications to standard right colectomy in experienced hands, with a survival signal favoring, although the strength of this evidence remains limited by study design (27). A 2025 SAGES/ESCP-supported systematic review and meta-analysis found no difference in 30-day mortality or major morbidity versus standard resection, while indicating possible gains in OS and DFS; certainty was limited by observational designs and heterogeneity (35). Seow-En et al. reviewed CME with D3 dissection and similarly reported no consistent increase in postoperative complications (despite acknowledged vascular-injury risk) and described a caudal-to-cranial dissection along the fully exposed superior mesenteric vessels to achieve safe high ligation (28). Mazzarella et al. reported promising oncologic signals (survival and recurrence), albeit with heterogeneous techniques and study quality (36). Indeed, the authors highlighted the lack of evidence on the CME learning curve, suggesting that outcomes may vary significantly between high-volume and low-volume centers.

**Figure 1:**
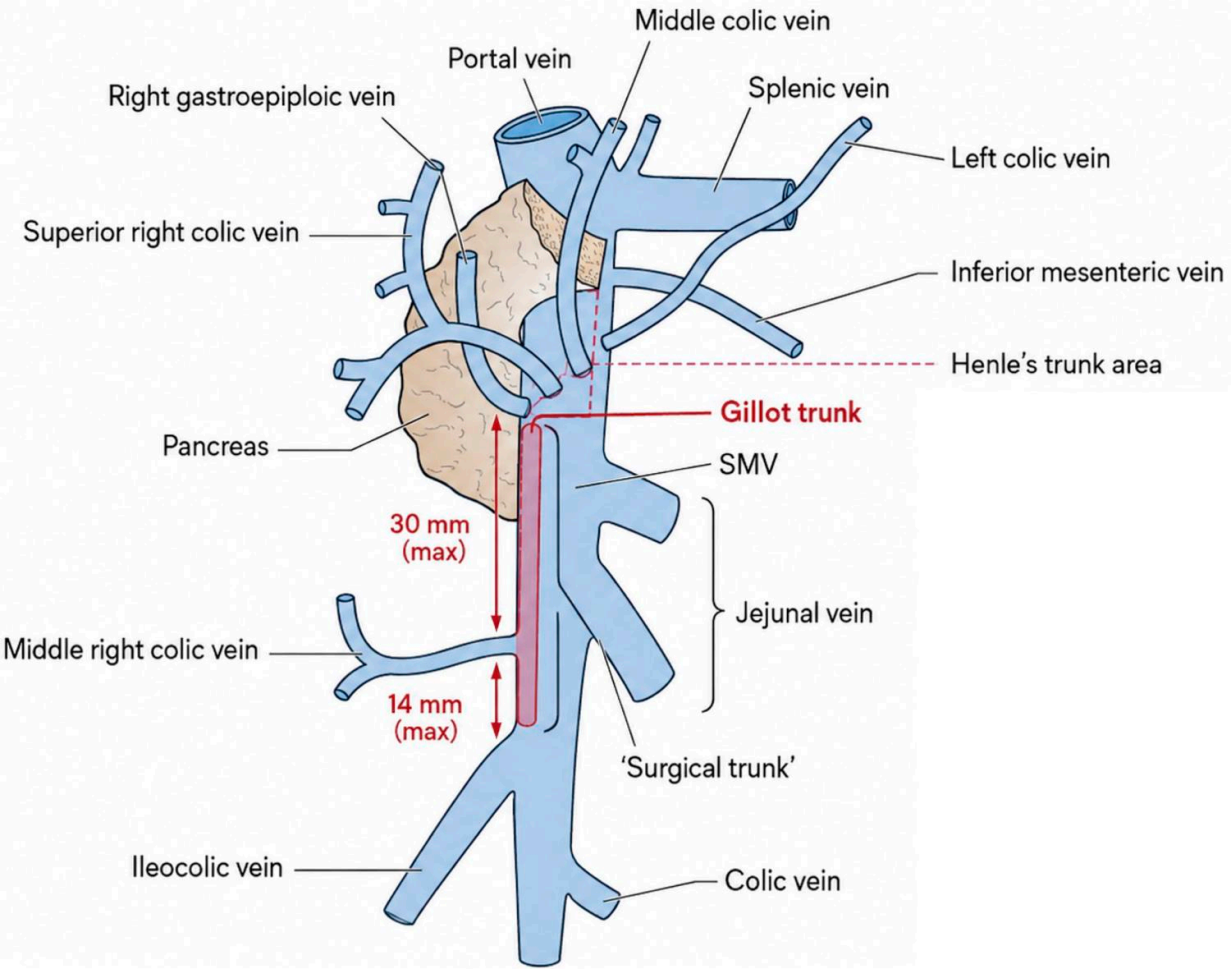
Anatomy of the mesenteric venous confluence highlighting the gillot trunk.

Several quantitative reviews support a staging and possibly survival advantage with more radical lymphadenectomies. Balciscueta et al. (EJSO) found that CME plus D3 was associated with longer resection length, greater vessel-to-tumor distance, larger mesenteric area, and higher lymph-node yield, with reductions in local recurrence and improved OS/DFS, particularly in stage III but with signals also in selected stage II tumors (37). Kong et al. concluded that CME reduces local and distant recurrence and improves 5-year OS versus conventional resection, but at the cost of higher vascular-injury risk (e.g., SMV injury with potential ischemia, ligation at the ileocolic origin, injury to Henle's trunk or the middle colic vein) (38). Using restricted-mean survival time, Aiolfi et al. estimated a modest average survival gain at 60 months for stages I–III (2.5 months) that was larger in stage III (6.1 months), emphasizing potential confounding by tumor biology and adjuvant therapy (39). Consistently, methodologic reviews note improved specimen-quality metrics and nodal yield with CME but highlight selection bias, heterogeneity in definitions, and the need for standardized reporting and audited adoption (40). Particularly, discrepancies are noted between Western “extended lymphadenectomy” and Eastern “D3”. According to the JSCCR definition, D3 lymphadenectomy includes dissection of the lymphatic tissue along both the lateral and medial aspects of the superior mesenteric vein, including the gastrocolic trunk of Henle, up to the root of the superior mesenteric artery. Many Western surgeons, however, favor a dissection along the superior mesenteric vein (the surgical trunk of Gillot) combined with central vascular ligation, without performing a formal lymphadenectomy around the root of the superior mesenteric artery. This dissection may satisfy the criteria of both CME and CVL, but not those of a D3 dissection according to the Japanese guidelines. Comprehension of anatomical landmarks that define the boundary between a D2 and D3 dissection (e.g., the exact relation to the SMA/SMV for right-sided tumors) will ensure universal understanding (40) (Figure 1).

Taken together, CME and CVL reliably increase nodal yield and resection radicality, which strengthens staging and may facilitate appropriate adjuvant therapy, with apparent oncologic benefits across stages II–III and the most consistent survival signal in stage III disease (41).

### Transverse and left-sided colon cancer

The evidence base for CME in transverse and left-sided resections is smaller, and oncologic principles are extrapolated from right-sided CME and from TME for rectal cancer. For distal transverse tumors, left colectomy with modified CME appears oncologically safe, with very low rates of nodal recurrence around the superior mesenteric vessels, which represent an unusual site of nodal dissemination in this setting (42). For splenic-flexure cancers, CME with central ligation is feasible and safe in experienced units, but central nodal metastases at the IMA level are uncommon, and long-term outcomes are favorable even without routine D3 dissection (43–45). Mid-transverse tumors often lie at the interface between right colic, middle colic, and left colic inflow, and may therefore require tailored extended right or left colectomies with a wider mesenteric root to encompass both arterial territories and their respective lymphatic drainage. In these patients, pre-operative vascular mapping and intra-operative assessment are key to choosing between segmental transverse colectomy and extended resections, aiming to achieve oncologic clearance while limiting unnecessary bowel sacrifice. The 2022 ASCRS guideline emphasizes that transverse-colon and splenic-flexure cancers require individualized consideration of resection type and lymphadenectomy extent, given variable vascular anatomy and mixed available data (11). Providing evidence-based definitions of D2 and D3 dissections for transverse and descending colon cancer is challenging because of the limited literature specifically addressing these anatomical locations.

In transverse colon cancer, the distinction mainly relates to the extent of dissection along the middle colic vessels and, when appropriate, towards the superior mesenteric vascular axis. In descending colon cancer, the difference lies in whether the dissection extends to the origin of the inferior mesenteric artery. Brown et al. (46) support the concept that a more extended lymphadenectomy on the left side should reach the origin of the inferior mesenteric artery. Similarly, Vogelsang et al. (47) suggested that the length of the inferior mesenteric artery stump may represent an objective indicator of the quality of central vascular ligation. The oncological benefit of such an extended dissection, however, remains a matter of debate also for these locations. Planellas et al. (48) did not demonstrate a clear oncological advantage of extended CME compared with standard resection, despite the performance of a more radical lymphadenectomy.

For left sided colon cancer, most surgeons perform IMA ligation near its origin (D2-level lymphadenectomy) during sigmoid or anterior resections. CME with CVL increases vessel-to-tumor distance, margin length, and mesenteric surface, but routine D3 lymphadenectomy is not universally supported in the absence of proven oncologic advantage, especially given the potential impact on autonomic nerves and genitourinary function (46). Objective measures of “how central” the tie is have been explored: on routine follow-up CT, an IMA arterial stump length ≥10 mm (versus <10 mm) correlated with several operative and pathological quality parameters. CT-based stump measurements may serve as a quality indicator, though their direct impact on survival is uncertain (47). Pathologic plane assessment after left-sided CME is also nuanced. In a cohort of stage I–III sigmoid cancers, an intact mesocolic plane did not translate into lower recurrence compared with intramesocolic plane dissection, suggesting plane grading alone is an unreliable prognostic parameter in this setting (49). In the only multicentre randomized trial of extended versus standard CME for sigmoid cancer, Planellas et al. showed no increase in total lymph nodes retrieved with the extended approach, no improvement in DFS/OS, and similar perioperative morbidity. Extended CME was associated with worse postoperative urinary function in men on follow-up analyses (48). The authors concluded to reserve this procedure for selected patients with evidence of pathological lymphadenopathy. In comparative series, robotic and laparoscopic left colectomy performed with CME achieved similar oncologic radicality and safety; robotic procedures yielded higher nodal counts but longer operating times and higher costs, without survival advantages (50).

However, the debate between high tie and low tie ligation of the inferior mesenteric artery in sigmoid resections remains controversial, mainly because the strongest evidence from randomized clinical trials has largely been derived from rectal cancer surgery. Clinical studies also addressing sigmoid resections are heterogeneous, often comprising also patients with rectal, rectosigmoid junction, and sigmoid cancers. High ligation would be associated with the benefits of maximum lymph nodal clearance and easy mobilization of the splenic flexure. However, drawbacks as potential nerves injury, compromised blood supply, and vascular problems in case of redo surgery have also been described. Conversely, this risk is minimal with low ligation, which is associated with suboptimal lymph nodal clearance and no mobilization of the splenic flexure.

Kim et al. (51) reported no significant differences between the high and low ligation techniques in terms of survival, disease recurrence, or postoperative complications, while highlighting a potential negative impact of high tie on bowel and sexual function. Yin et al. (52) demonstrated that low tie provides oncological outcomes comparable to those of high tie and is associated with a significantly lower risk of anastomotic leakage. More recently, You et al. (53), in a meta-analysis including randomized controlled trials, confirmed that low tie is associated with a lower incidence of anastomotic leakage and faster postoperative recovery, without compromising lymph node harvest, recurrence rates, disease-free survival, or overall survival.

Globally, the currently available evidence does not support the oncological superiority of high tie over low tie. However, since most high-quality randomized evidence derives from rectal cancer surgery, further studies are needed before definitive conclusions can be drawn regarding sigmoid colon resections.

*Practical take-home message: CME, CVL, and D3 lymphadenectomy should be regarded as components of high-quality colon cancer surgery that can enhance specimen quality, nodal clearance, and staging accuracy. Their adoption is most strongly supported for stage III and high-risk stage II disease, particularly in right-sided tumors, and should be implemented through structured training and continuous auditing of perioperative and oncologic outcomes rather than being restricted to a few expert centers.*

## Conclusion

**Figure 2:**
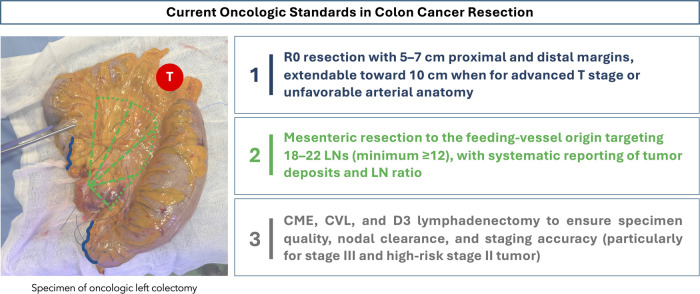
Current oncologic standards in colon cancer resection. Specimen of oncologic left colectomy (on the right) and summary of the main oncological principles of colon cancer resection. (Blue lines on the surgical specimen indicate proximal and distal margins; dotted green lines identify mesentery with feeding-vessels; T indicates tumor location).

Oncologic adequacy in contemporary colon cancer surgery is multidimensional. Oncological standards should be balanced between oncologic radicality and the preservation of autonomic nerves/functional quality of life. The preponderance of evidence supports 5–7 cm longitudinal margins for most tumors, extending toward 10 cm for advanced T stage or unfavorable arterial anatomy, provided perfusion and function are maintained. LN yield should exceed the historical minimum, aiming for 18–22 nodes to improve staging precision and oncological outcomes, while pathology should systematically report tumor deposits and lymph node ratio. CME, CVL, and D3 augment specimen quality and may improve oncologic outcomes across stages II–III, with the clearest survival signal in stage III disease, but their benefits remain contingent on surgeons' experience, training, and thoughtful patient selection (Figure 2). Rather than restricting these techniques to a few expert centers, they should be progressively integrated into standard colon cancer surgery through structured training, proctored implementation, and continuous auditing of short- and long-term outcomes. Future studies should standardize definitions, stratify by colon segment and molecular risk, and embed ctDNA endpoints to disentangle surgical from biologic effects.
